# Research on Digital Terrain Construction Based on IMU and LiDAR Fusion Perception

**DOI:** 10.3390/s25010015

**Published:** 2024-12-24

**Authors:** Chen Huang, Yiqi Wang, Xiaoqiang Sun, Shiyue Yang

**Affiliations:** 1 Institute of Automotive Engineering, Jiangsu University, Zhenjiang 212013, China; huangchen@ujs.edu.cn (C.H.); sunxqujs@126.com (X.S.); yangshiyue99@163.com (S.Y.); 2 National Key Laboratory for Intelligent and Green Vehicles and Transportation, Tsinghua University, Beijing 100084, China

**Keywords:** terrain preview, road surface elevation information, front tire trajectory, moving average-like algorithm

## Abstract

To address the shortcomings of light detection and ranging (LiDAR) sensors in extracting road surface elevation information in front of a vehicle, a scheme for digital terrain construction based on the fusion of an Inertial Measurement Unit (IMU) and LiDAR perception is proposed. First, two sets of sensor coordinate systems were configured, and the parameters of LiDAR and IMU were calibrated. Then, a terrain construction system based on the fusion perception of IMU and LiDAR was established, and improvements were made to the state estimation and mapping architecture. Terrain construction experiments were conducted in an academic setting. Finally, based on the output information from the terrain construction system, a moving average-like algorithm was designed to process point cloud data and extract the road surface elevation information at the vehicle’s trajectory position. By comparing the extraction effects of four different sliding window widths, the 4 cm width sliding window, which yielded the best results, was ultimately selected, making the extracted road surface elevation information more accurate and effective.

## 1. Introduction

With the continuous advancement of vehicle suspension technology, intelligent suspension systems are capable of autonomously sensing the terrain ahead, making decisions, and executing complex suspension control tasks [1,2]. The realization of this capability relies heavily on sensor technology. As an indispensable component of intelligent driving systems, sensor technology provides real-time and precise environmental information for intelligent suspension systems. However, the performance of the intelligent suspension system is directly affected by the accuracy of the sensors and the quality of the algorithms [3,4]. With the ongoing development of technologies such as LiDAR and visual sensors, it has become possible to accurately acquire elevation information for the road surface in front of the vehicle. However, research on acquiring elevation information for the road surface in front of the vehicle is currently limited. Therefore, it is necessary to conduct digital terrain research on front road surface perception.

For terrain-detection technologies [5,6,7,8,9], researchers are utilizing sensor technologies for terrain measurement, which can be mainly divided into three research directions: vision-based terrain-detection methods, LiDAR-based terrain-detection methods, and terrain-detection methods based on multi-sensor fusion. Vision-based terrain-detection methods have advantages such as a low cost and low energy consumption, which have led many scholars to conduct extensive research on map construction based on visual sensors. Klein et al. [10] utilized the concepts of vision and keyframes to enhance the real-time capability of mapping, although this method is primarily applicable for map construction and navigation, making it inadequate for high-precision mapping needs. Raul et al. [11] proposed a vision-based map construction algorithm that simultaneously processes feature detection and tracking, local map construction, and loop-closure detection optimization, thereby improving the accuracy and robustness of the algorithm to achieve high-precision positioning and navigation. However, this method struggles to acquire elevation information for the road surface in front of the vehicle. Additionally, since visual sensors are sensitive to lighting, texture, and rapid motion, their application scenarios may be limited [12,13,14,15]. Thus, vision-based terrain-detection methods are mainly applied in indoor environments and relatively structured roadways.

LiDAR-based terrain-detection methods offer advantages such as long measurement distances, high ranging accuracy, high resolution, and immunity to lighting conditions. Consequently, LiDAR is more advantageous for identifying road surface information. Zhang et al. [16] proposed the real-time LiDAR Odometry and Mapping (LOAM) algorithm, which enables real-time odometry and mapping functions using LiDAR. However, this algorithm lacks loop-closure detection and cannot handle large-scale rotational transformations. Shan et al. [17] extended the LOAM algorithm to develop the Lightweight and Ground-Optimized LiDAR Odometry and Mapping on Variable Terrain (LeGO-LOAM) algorithm. This method optimizes the algorithm’s lightweight nature and ground adaptability through point cloud classification and feature extraction. It also incorporates loop-closure detection and pose graph optimization, enhancing the system’s robustness. Nevertheless, the ground point extraction algorithm of this method does not meet the needs of complex terrain and requires a precise LiDAR installation. To reduce drift errors during the mapping process, Lin et al. [18] proposed a loop-closure detection-based algorithm to correct accumulated errors in the map. This method calculates the 2D histograms of keyframes and uses Normalized Cross-Correlation (NCC) as a similarity metric between adjacent keyframes. Experimental results show that this approach improves the reliability and robustness of the algorithm. Wang et al. [19] introduced a lightweight 3D localization and mapping method for solid-state LiDAR, named Solid-State LiDAR Simultaneous Localization and Mapping (SSL SLAM). Building on previous research, this method employs a scan-to-map approach to enhance performance and a sliding window technique to reduce computational costs, ensuring real-time capability. With the ongoing enhancement of LiDAR performance, high-beam LiDARs can extract clearer contour information. As LiDARs are mass-produced, their costs will continue to decrease. Therefore, LiDAR technology is advancing towards higher performance, lower cost, and broader applications to meet market demands in various fields [20,21,22].

Terrain-detection methods based on multi-sensor fusion are more commonly used. This approach leverages the advantages of combining multiple sensors to compensate for the shortcomings of individual sensors, thereby improving data accuracy and robustness, and achieving more reliable terrain analysis and technical support in complex and variable environments. Nguyen-Ngoc et al. [23] proposed a tightly coupled GPS/INS/LIDAR integration system that uses Factor Graph Optimization (FGO) to integrate measurement data from various sensors into the system, enabling high-precision localization and mapping. However, the multi-sensor fusion system faces challenges related to the time synchronization and spatial calibration of different sensors, as well as high computational resource requirements, and the algorithm suffers from error accumulation issues. Therefore, further improvements are needed in terms of real-time performance and robustness. Almalioglu et al. [24] developed a framework based on the Unscented Kalman Filter (UKF) that integrates millimeter-wave radar and IMU for low-cost indoor 6DoF pose estimation. However, the accuracy, resolution, and processing speed of the millimeter-wave radar used are difficult to match with those of LiDAR. Su et al. [25] proposed the LiDAR-based sensor fusion SLAM for ground robots on complex terrain (GR-LOAM) algorithm, which achieves high precision and robustness in positioning and mapping for ground robots on complex terrain by fusing LiDAR, IMU, and encoder data. This method has high demands for real-time performance and accuracy, but there is no in-depth study on the road elevation information. Shan et al. [26] proposed the Tightly Coupled LiDAR Inertial Odometry via Smoothing and Mapping (LIO-SAM) algorithm, which is a tightly coupled SLAM algorithm based on LiDAR and IMU and an extension of LeGO-LOAM. This framework achieves tightly coupled LiDAR-inertial odometry through smoothing and mapping by optimizing the LiDAR odometry factors, IMU pre-integration factors, GPS factors, and loop-closure detection factors to obtain globally consistent pose estimates, thus improving the system accuracy and robustness. In summary, for extracting road elevation information in complex terrain, road perception systems can effectively reduce extraction errors and improve the system accuracy and robustness not only through multi-sensor fusion methods, but also through improved ground point extraction algorithms. Based on the issues identified in the above research, this paper selects high-precision LiDAR to reduce measurement errors in road elevation information systems. Additionally, an improved framework for road point cloud mapping is proposed to obtain high-precision road point cloud information. On this basis, a moving average-like algorithm is proposed to balance both accuracy and efficiency, ensuring that the extracted road elevation information is more precise and effective.

The main contributions of this paper are summarized as follows:

(1) A terrain construction system based on the fusion of IMU and LiDAR perception is established. This system includes the architecture for state estimation and mapping, IMU pre-integration factors, LiDAR odometry factors, and loop-closure detection factors.
(2) Considering the large computational load of the original architecture, this paper improves the existing terrain construction system by adding a new functional module to the algorithm framework. This module saves each point cloud frame with complete road surface details and stitches multiple point clouds together to generate a point cloud file containing the complete road surface information.
(3) A new moving average-like algorithm is proposed to address the challenges in extracting road elevation information. By comparing the extraction effects of sliding windows with different widths, the optimal window width is determined, thereby improving the accuracy of road elevation information extraction.

The remainder of the paper is organized as follows: Section 2 describes the configuration of the sensor coordinate systems and completes the parameter calibration between the LiDAR and IMU. Section 3 builds a terrain construction model based on the fusion of IMU and LiDAR perception. Building on this model, improvements are made to the state estimation and mapping architecture, and terrain construction experiments are conducted at a school facility. Section 4 utilizes the point cloud library to extract point cloud information about the front tire trajectory, and designs moving average-like algorithm to determine the optimal sliding window width for extraction effectiveness. By comparing it with the Gaussian filter algorithm, the superiority of the moving average-like algorithm is validated. Section 5 concludes the paper.

## 2. Sensor Coordinate Systems and Parameter Calibration

### 2.1. Sensor Coordinate System Configuration

**Figure 1:**
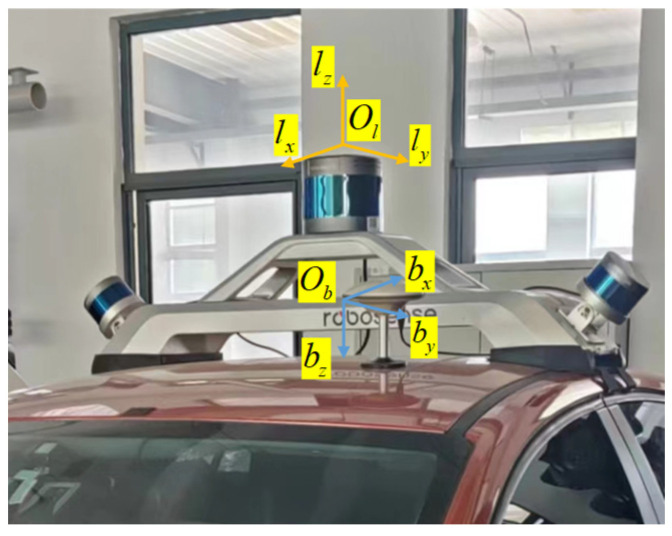
LiDAR coordinate system and IMU coordinate system.

The terrain construction system contains two main coordinate systems: the LiDAR coordinate system and the IMU coordinate system. Figure 1 illustrates the relationship between the LiDAR coordinate system and the IMU coordinate system.

(1) LiDAR coordinate system

Taking the geometric center of the LiDAR device as the origin, a right-handed coordinate system is employed where the vehicle’s longitudinal forward direction is the *x*-axis, the horizontal left direction is the *y*-axis, and the vertical upward direction is the *z*-axis. The coordinate system is denoted by *l*, with the axes labeled as $O_{l}-l_{x}l_{y}l_{z}$.

(2) IMU coordinate system

The IMU coordinate system is considered the body coordinate system, using a right-handed coordinate system where the vehicle’s longitudinal backward direction is the *x*-axis, the horizontal left direction is the *y*-axis, and the vertical downward direction is the *z*-axis. The coordinate system is denoted by *b*, with the axes labeled as $O_{b}-b_{x}b_{y}b_{z}$.

### 2.2. Sensor Parameters Calibration

The calibration between the LiDAR and the IMU is aimed at eliminating errors and discrepancies between them. Through calibration, targets in the LiDAR coordinate system can be transformed into the corresponding body coordinate system of the IMU, ensuring the accuracy and consistency of measurement results [27]. Specifically, the following objectives can be achieved:

(1) Coordinate System Alignment

LiDAR and IMU each have their own coordinate systems. Through calibration, the transformation relationship between them can be determined, thereby allowing their measurement results to be compared and analyzed in the same coordinate system.

(2) Angle Compensation

The measurement results of the IMU may be subject to interference from factors such as vibrations and acceleration, leading to errors in angle measurement. By calibrating with the LiDAR, the angular measurement errors of the IMU can be accurately compensated, thereby improving the accuracy of attitude estimation.

(3) Position Calibration

There may be minor installation errors in the positions of the LiDAR and IMU, leading to slight discrepancies in their spatial locations. Calibration can determine the positional offsets between them, allowing for the adjustment of their location data and thereby improving the accuracy of positioning.

**Figure 2:**
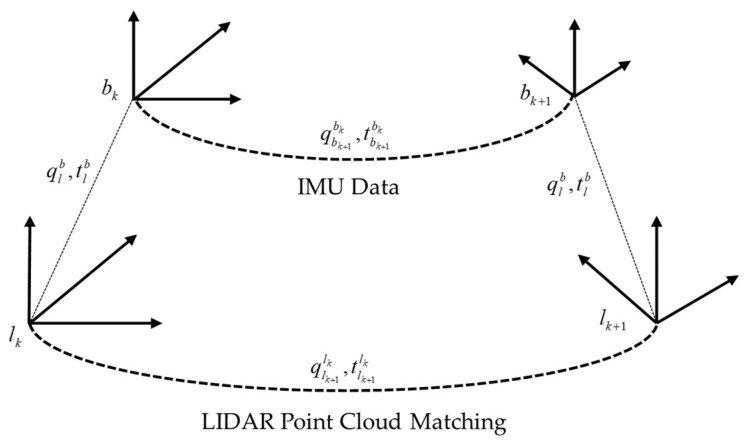
LiDAR and IMU calibration diagram.

The relationship between the LiDAR and IMU is solved through the loosely coupled approach using IMU pre-integration and LiDAR odometry, as illustrated in Figure 2. For two keyframes *k* and *k* + 1, the rotation matrix obtained from the IMU pre-integrating between the frames is $q_{b_{k+1}}^{b_{k}}$, while the relative rotation computed from the LiDAR odometry is $q_{l_{k+1}}^{l_{k}}$. Since both describe the same rotation, the following constraint relationship [28] holds:

(1) $$\begin{array}{c} q_{b_{k+1}}^{b_{k}}q_{l}^{b}=q_{l}^{b}q_{l_{k+1}}^{l_{k}} \end{array}$$

To facilitate calculations, this paper introduces the left and right multiplication matrices for quaternions. For a quaternion $q={\left[s,\;v\right]}^{T}$ in Hamilton form, the following matrices are used:

(2) $$\begin{array}{c} {\left[q\right]}_{L}=\left[\begin{array}{cc} s & -v^{T}\\ v & sI+{\left[v\right]}_{x} \end{array}\right],{\left[q\right]}_{R}=\left[\begin{array}{cc} s & -v^{T}\\ v & sI-{\left[v\right]}_{x} \end{array}\right] \end{array}$$

where *s* is the scalar part and *v* is the vector part of the quaternion.

To convert the quaternion representation into matrix operations, the constraints can be expressed as follows:

(3) $$\begin{array}{c} \left({\left[q_{b_{k+1}}^{b_{k}}\right]}_{L}-{\left[q_{l_{k+1}}^{l_{k}}\right]}_{R}\right)q_{l}^{b}=0 \end{array}$$

Therefore, a set of linear equations can be constructed using a group of keyframes, typically choosing 10 to 20 keyframes to form an overdetermined system. By solving the least squares problem, the extrinsic rotation parameters represented by quaternions can be obtained. Since obtaining precise results for translation vectors is challenging, manual measurement is often used to determine them.

## 3. Ground Construction Model Based on Sensor Fusion

### 3.1. State Estimation and Mapping Architecture

First, we define the coordinate systems and symbols used for vehicle state estimation and mapping. The coordinate system *w* is defined as the world coordinate system, constructed with the origin of the coordinate system *b* from the first set of keyframes received as the starting point. The vehicle’s driving state will be represented in the world coordinate system *w*. The IMU is closely connected to the vehicle, so the coordinate system of the IMU perfectly overlaps with the vehicle’s coordinate system. Therefore, the transformation matrix between them is the identity matrix. The system state *x* of the vehicle can be expressed as follows:

(4) $$\begin{array}{c} x={\left[R^{T},p^{T},v^{T},b^{T}\right]}^{T^{\prime }} \end{array}$$

where *R* is the rotation matrix, *p* is the position vector, *v* is the velocity, *b* is the IMU bias, and T is the transpose. The transformation matrix $T^{\prime }$ represents the transformation from the vehicle coordinate system to the global coordinate system, given by

(5) $$\begin{array}{c} T^{\prime }=\left[R|P\right] \end{array}$$

**Figure 3:**
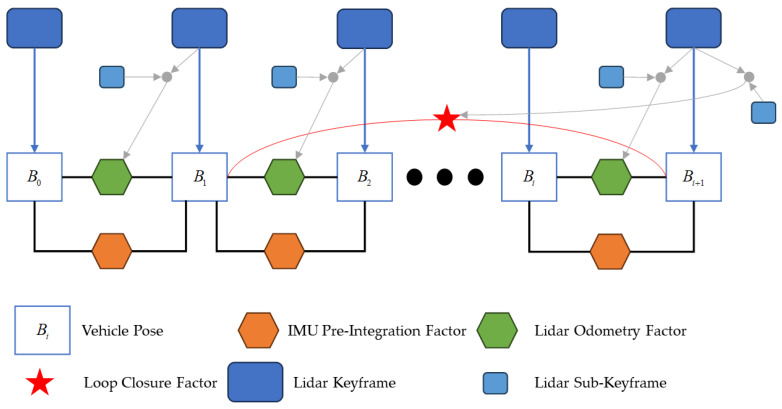
Overall architecture of state estimation.

The overall architecture of state estimation is shown in Figure 3.

The system receives sensor data from LiDAR and IMU and estimates the vehicle’s state based on the observations from these sensors. This paper uses a factor graph to model the problem, as it is better suited for inference compared to Bayesian networks. Under the assumption of Gaussian noise models, the overall architecture can be represented using the following nonlinear least squares optimization, aiming to minimize the total residual of the system by solving for the state variables:

(6) $$\begin{array}{c} X^{*}=\arg\underset{X}{min}\;F(\overset{\wedge }{z},X) \end{array}$$

(7) $$\begin{array}{c} F\left(\overset{\wedge }{z},X\right)=\sum_{k\in B}{\left\|r_{b}({\overset{\wedge }{z}}_{b_{k+1}}^{b_{k}},X)\right\|}_{\sum \begin{array}{c} b_{k}\\ b_{k+1} \end{array}}^{2}+\sum_{k\in L}{\left\|r_{l}({\overset{\wedge }{z}}_{l_{k+1}}^{l_{k}},X)\right\|}_{\sum \begin{array}{c} l_{k}\\ l_{k+1} \end{array}}^{2} \end{array}$$

where *X* denotes all the state variables to be optimized in the factor graph, and $\hat{z}$ represent all the observations. $r\left(\cdots \right)$ is the residual computation function for the corresponding constraints, and ${\left\|\cdots \right\|}_{\Sigma }^{2}$ is the vector norm calculated using the covariance corresponding to the constraints. *B* and *L* represent the constraint observations from the IMU and LiDAR, respectively.

### 3.2. IMU Pre-Integration Factor

The measurements of IMU acceleration and angular velocity are defined as follows:

(8) $$\begin{array}{c} a_{m}^{b}=R_{w}^{b}\left(a^{w}-g^{w}\right)+b_{a}+n_{a} \end{array}$$

(9) $$\begin{array}{c} \omega_{m}^{b}=\omega^{b}+b_{g}+n_{g} \end{array}$$

where $a_{m}^{b}$ and $\omega_{m}^{b}$ are the raw acceleration and angular velocity data measured by the IMU in the body coordinate system, respectively. $g^{w}$ denotes the gravitational acceleration in the world coordinate system. $n_{a}$ and $n_{g}$ are the acceleration noise and angular velocity noise of the IMU during measurement, respectively. $b_{a}$ and $b_{g}$ are the acceleration bias and angular velocity bias of the IMU, respectively. $R_{w}^{b}$ is the rotation matrix from the world coordinate system to the body coordinate system.

After optimizing the state variables, the system predicts the current latest state using the data obtained from the IMU measurements. In this paper, the state variables at the *k*-th keyframe are defined as $x_{b_{k}}^{w}$. The IMU model is expressed as follows:

(10) $$\begin{array}{c} p_{b_{k+1}}^{w}=p_{b_{k}}^{w}+v_{b_{k}}^{w}\Delta t+\frac{1}{2}g^{w}{\Delta t}^{2}+R_{b_{k}}^{w}\iint_{t_{k}}^{t_{k+1}}R_{b_{t}}^{b_{k}}\left(a_{m_{t}}^{b}-b_{a_{t}}-n_{a}\right){dt}^{2} \end{array}$$

(11) $$\begin{array}{c} v_{b_{k+1}}^{w}=v_{b_{k}}^{w}+g^{w}\Delta t+R_{b_{k}}^{w}\int_{t_{k}}^{t_{k+1}}R_{b_{t}}^{b_{k}}\left(a_{m_{t}}^{b}-b_{a_{t}}-n_{a}\right)dt \end{array}$$

(12) $$\begin{array}{c} q_{b_{k+1}}^{w}=q_{b_{k}}^{w}⨂\int_{t_{k}}^{t_{k+1}}q_{b_{t}}^{b_{k}}⨂\left(\omega_{m_{t}}^{b}-b_{g_{t}}-n_{g}\right)dt \end{array}$$

Using the model from the above equation, the velocity, position, and attitude of the body at the *k* + 1-th frame can be estimated based on the velocity, position, and attitude of the body at the *k*-th frame. Since the time difference Δ*t* between the two keyframes is very small, the acceleration bias and angular velocity bias can be neglected.

For computational convenience, this paper uses the midpoint method to discretize the integration over continuous time. Specifically, the state propagation is carried out by averaging the IMU data at time *i* and the IMU data at the adjacent time *j*.

(13) $$\begin{array}{c} \bar{a}=\frac{1}{2}\left(R_{b_{i}}^{w}\left(a_{m_{i}}^{b}-b_{a}\right)+R_{b_{j}}^{w}\left(a_{m_{j}}^{b}-b_{a}\right)\right)+g^{w} \end{array}$$

(14) $$\begin{array}{c} \bar{\omega }=\frac{1}{2}\left(\omega_{m_{i}}^{b}+\omega_{m_{j}}^{b}\right)-b_{g} \end{array}$$

where $\bar{a}$ and $\bar{\omega }$ represent the average of the IMU data at time *i* and the IMU data at the adjacent time *j*. Substituting these values into the continuous-time IMU model yields the discretized IMU motion model as follows:

(15) $$\begin{array}{c} p_{b_{j}}^{w}=p_{b_{i}}^{w}+v_{b_{i}}^{w}\Delta t+\frac{1}{2}\bar{a}\delta t^{2} \end{array}$$

(16) $$\begin{array}{c} v_{b_{j}}^{w}=v_{b_{i}}^{w}+\bar{a}\delta t \end{array}$$

(17) $$\begin{array}{c} q_{b_{j}}^{w}=q_{b_{i}}^{w}⨂\left[\begin{array}{c} 1\\ \frac{1}{2}\bar{\omega }\delta t \end{array}\right] \end{array}$$

To address the real-time issues associated with re-integration when updating state variables after optimization, this paper adopts a pre-integration approach. This method treats the IMU data between adjacent keyframes as an independent factor, unrelated to changes in other state variables, and only dependent on the acceleration and angular velocity biases of the IMU. This achieves decoupling from the state variables. By doing so, it avoids the time delay caused by re-integration after updating the state variables, improving the system’s real-time performance. By constructing independent IMU factors, state updates and integration processes can be handled more flexibly, making the system more stable and efficient during operation. Decoupling the state variable $x_{b_{k}}^{w}$ from the continuous-time IMU model yields

(18) $$\begin{array}{c} \Delta p_{b_{k+1}}^{b_{k}}=\iint_{t_{k}}^{t_{k+1}}R_{b_{t}}^{b_{k}}\left(a_{m_{t}}^{b}-b_{a_{t}}-n_{a}\right){dt}^{2} \end{array}$$

(19) $$\begin{array}{c} \Delta v_{b_{k+1}}^{b_{k}}=\int_{t_{k}}^{t_{k+1}}R_{b_{t}}^{b_{k}}\left(a_{m_{t}}^{b}-b_{a_{t}}-n_{a}\right)dt \end{array}$$

(20) $$\begin{array}{c} \Delta q_{b_{k+1}}^{b_{k}}=\int_{t_{k}}^{t_{k+1}}q_{b_{t}}^{b_{k}}⨂\left(\omega_{m_{t}}^{b}-b_{g_{t}}-n_{g}\right)dt \end{array}$$

where $\Delta p_{b_{k+1}}^{b_{k}}$, $\Delta v_{b_{k+1}}^{b_{k}}$, and $\Delta q_{b_{k+1}}^{b_{k}}$ are the pre-integrated quantities for position, velocity, and attitude, respectively, between the *k*-th frame and *k* + 1-th frame. At this point, because the pre-integrated quantities are in the body coordinate system, they do not change due to updates in the state variables. After discretizing the pre-integrated IMU quantities using the midpoint method, the pre-integration of the IMU at time *i* and the adjacent time *j* is given by

(21) $$\begin{array}{c} \Delta p_{b_{j}}^{b_{k}}=\Delta p_{b_{i}}^{b_{k}}+{\Delta v}_{b_{i}}^{b_{k}}\delta t+\frac{1}{2}\bar{a}\delta t^{2} \end{array}$$

(22) $$\begin{array}{c} \Delta v_{b_{j}}^{b_{k}}={\Delta v}_{b_{i}}^{b_{k}}+\bar{a}\delta t \end{array}$$

(23) $$\begin{array}{c} \Delta q_{b_{j}}^{b_{k}}=\Delta q_{b_{i}}^{b_{k}}⨂\left[\begin{array}{c} 1\\ \frac{1}{2}\bar{\omega }\delta t \end{array}\right] \end{array}$$

where

(24) $$\begin{array}{c} \bar{a}=\frac{1}{2}\left(R_{b_{i}}^{b_{k}}\left(a_{m_{i}}^{b}-b_{a}\right)+R_{b_{j}}^{b_{k}}\left(a_{m_{j}}^{b}-b_{a}\right)\right) \end{array}$$

(25) $$\begin{array}{c} \bar{\omega }=\frac{1}{2}\left(\omega_{m_{i}}^{b}+\omega_{m_{j}}^{b}\right)-b_{g} \end{array}$$

### 3.3. LiDAR Odometer Factor

When a new LiDAR frame is received, feature extraction is first performed. Edge and plane features are extracted by evaluating the roughness of points in a local region. Points with higher roughness are classified as edge features, while points with lower roughness are classified as plane features. At time *i*, the edge and plane features extracted from the LiDAR scan are defined as $F_{i}^{e}$ and $F_{i}^{p}$, respectively. All the features extracted at time *i* constitute the LiDAR frame $F_{i}$, which is located in the body coordinate system, where

(26) $$\begin{array}{c} F_{i}=\left\{F_{i}^{e},F_{i}^{p}\right\} \end{array}$$

To avoid excessive computational load from adding every LiDAR frame to the factor graph, this paper uses a keyframe approach. When the vehicle’s pose changes exceed a predetermined threshold (displacement: 0.8 m, angle: 10°), $F_{i+1}$ is selected as a keyframe. The newly saved keyframe, $F_{i+1}$, is associated with the new vehicle state node in the factor graph, while LiDAR frames between two keyframes are ignored. This approach not only balances map density and memory consumption, but also helps maintain a relatively sparse factor graph. Suppose a new state node, $x_{i+1}$, is added to the factor graph, and its corresponding LiDAR keyframe is $F_{i+1}$. The steps to generate the LiDAR odometry factor are as follows:

Firstly, a point cloud map containing a fixed number of recent LiDAR scans is constructed, and *n* latest keyframes, referred to as sub-keyframes, are extracted for estimation. Then, using the transformation matrices $\left\{T_{i-n},\ldots ,T_{i}\right\}$ associated with the set of sub-keyframes $\left\{F_{i-n},\ldots ,F_{i}\right\}$, the sub-keyframes are transformed into the world coordinate system. The transformed sub-keyframes are merged to form a voxel map, $M_{i}$. Since two types of features, namely, edge features and plane features, were extracted in the previous feature extraction step, the voxel map $M_{i}$ consists of two sub-voxel maps, denoted as the edge feature voxel map, $M_{i}^{e}$, and the plane feature voxel map, $M_{i}^{p}$. The relationship between the LiDAR frame and the voxel map is expressed as follows:

(27) $$\begin{array}{c} M_{i}=\left\{M_{i}^{e},M_{i}^{p}\right\} \end{array}$$

where

(28) Mie=Fie′∪Fi−1e′∪…∪Fi−ne′

(29) Mip=Fip′∪Fi−1p′∪…∪Fi−np′

where Fie′ and Fip′ are edge features and plane features in the world coordinate system, respectively.

Next, for the newly received LiDAR frame, $F_{i+1}$, we match the scan with the voxel map $M_{i}$. We then transform the edge and plane features $F_{i+1}^{e}$ and $F_{i+1}^{p}$ extracted from the LiDAR scan from the body coordinate system to the world coordinate system to obtain Fi+1e′ and Fi+1p′, where the transformation matrix is ${\tilde{T}}_{i+1}$ from the IMU. Then, we find the corresponding edge and plane relationships in $M_{i}^{e}$ and $M_{i}^{p}$ with the features in Fi+1e′ and Fi+1p′. The distance between a feature and its corresponding edge or plane can be calculated using the following formula:

(30) $$\begin{array}{c} d_{e_{k}}=\frac{\left|(p_{i+1,k}^{e}-p_{i,u}^{e})\times (p_{i+1,k}^{e}-p_{i,v}^{e})\right|}{\left|p_{i,u}^{e}-p_{i,v}^{e}\right|} \end{array}$$

(31) $$\begin{array}{c} d_{p_{k}}=\frac{\left|\begin{array}{c} \left(p_{i+1,k}^{p}-p_{i,u}^{p}\right)\\ \left(p_{i,u}^{p}-p_{i,v}^{p})\times (p_{i,u}^{p}-p_{i,w}^{p}\right) \end{array}\right|}{\left|(p_{i,u}^{p}-p_{i,v}^{p})\times (p_{i,u}^{p}-p_{i,w}^{p})\right|} \end{array}$$

where *k*, *u*, *v*, and *w* are the indices of the feature point correspondences. $p_{i+1}^{e}$ is an edge feature point in Fi+1e′, while $p_{i,u}^{e}$ and $p_{i,v}^{e}$ are two points on the edge line corresponding to this edge feature point in $M_{i}$. For a plane feature point in Fi+1p′, the corresponding plane patch in $M_{i}$ is defined by three points: $p_{i,u}^{p}$, $p_{i,v}^{p}$, and $p_{i,w}^{p}$.

Finally, we use the Gauss–Newton (G-N) method to solve the following residual minimization problem to obtain the optimal transformation matrix [29]:

(32) minTi+1⁡∑pi+1,ke∈Fi+1e′dek+∑pi+1,kp∈Fi+1p′dpk

Subsequently, the relative transformation $\Delta T_{i,i+1}$ between $x_{i}$ and $x_{i+1}$ can be obtained using the following equation, which represents the LiDAR odometry factor connecting these two poses:

(33) $$\begin{array}{c} \Delta T_{i,i+1}=T_{i}^{T}T_{i+1} \end{array}$$

### 3.4. Loop-Closure Factor

In this paper, a loop-closure detection method [30,31] based on the Euclidean distance is used, where the index *m* is set to 15, and the search distance for the loop closure from the new state $x_{i+1}$ is set to 20 m. When a new state, $x_{i+1}$, is added to the factor graph, the graph is first searched and previous states close to $x_{i+1}$ in the Euclidean space are found. For example, for $x_{5}$, the submap $F_{i+1}$ is first transformed into the world coordinate system along with past keyframes, and then $F_{i+1}$ is scanned and matched with the keyframes $\left\{F_{5-m},\ldots ,F_{5},\ldots ,F_{5+m}\right\}$. Finally, the relative transformation, $\Delta T_{5,i+1}$, is added to the factor graph as a loop-closure factor.

### 3.5. Terrain Construction Experiment Based on an Improved Architecture

**Figure 4:**
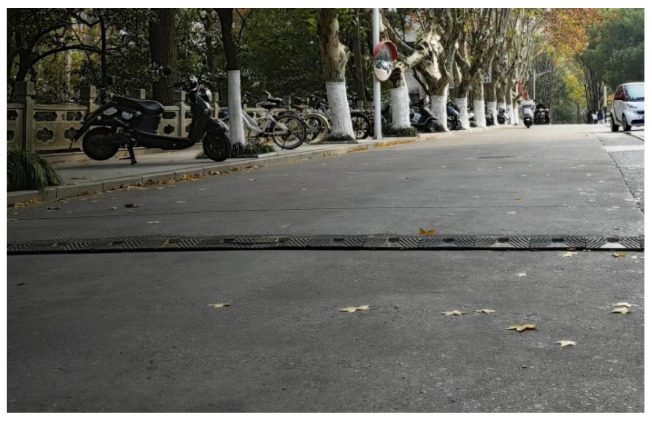
Road segment with speed bump on campus.

**Figure 5:**
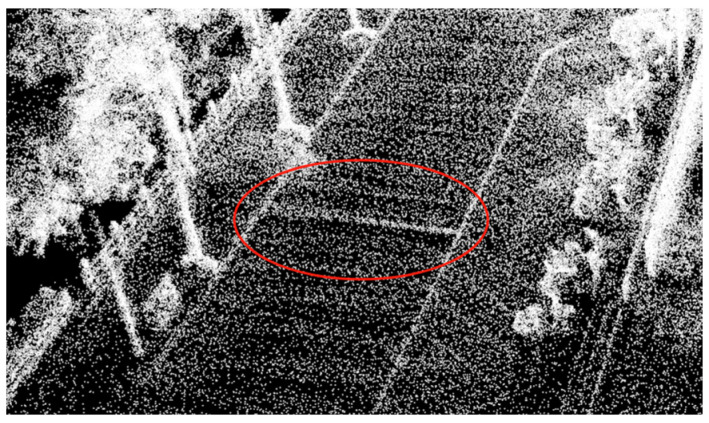
Point cloud map of road segment with speed bump.

To verify the effectiveness and accuracy of the terrain construction system, this paper established a Chery experimental vehicle platform equipped with an IMU and LiDAR sensors. The RS-Ruby Lite 80-line LiDAR was used to collect environmental point cloud data, while the 3DM-GX5-AHRS IMU provided motion and attitude information for the vehicle such as acceleration, angular velocity, and heading angle. A segment of a straight road within the campus featuring a speed bump was selected. The vehicle traveled along this road at a constant speed. During the vehicle’s movement, data collected using various sensors were input to the ECU and used for state estimation and mapping on the ROS platform. Figure 4 shows the road with the speed bump on the campus, and Figure 5 presents the point cloud map of the road with the speed bump.

From Figure 5, basic road surface information can be obtained, with the red circle highlighting the point cloud information of the speed bump. Since the architecture does not require detailed road surface information, a significant number of “irrelevant” planar points were filtered out during the mapping process to reduce data processing load. This, however, is clearly detrimental to the generation of road surface information.

**Figure 6:**
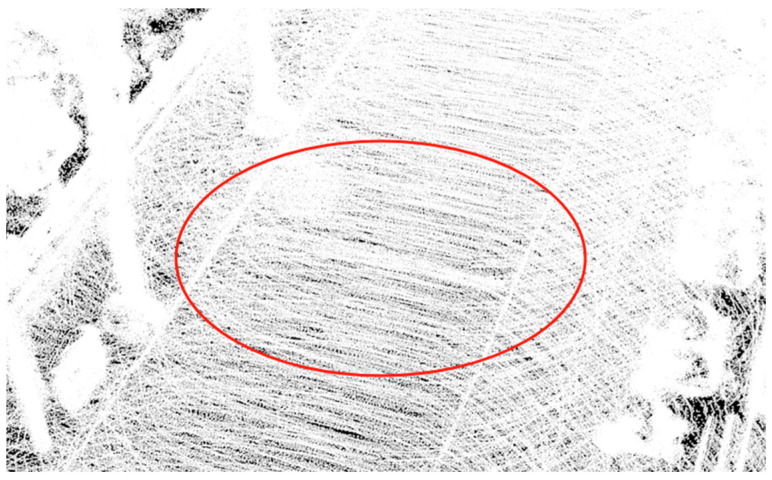
Point cloud map of road segment with speed bump generated using improved architecture.

To obtain detailed road surface point cloud data, this paper introduces a new functional module into the algorithmic architecture. This module saves each point cloud file with complete road surface details and stitches together multiple point clouds to create a single point cloud file with comprehensive road surface information. Basis on this, road surface elevation data were obtained. Figure 6 shows the point cloud map of the road segment with a speed bump generated using the improved architecture, with the red circle highlighting the point cloud information of the speed bump.

**Figure 7:**
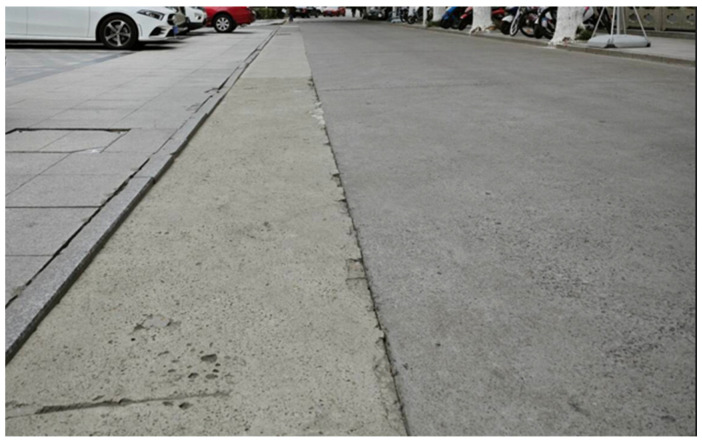
Uneven road surface used for the formal experiment.

**Figure 8:**
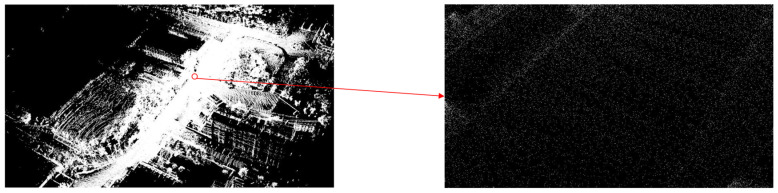
Point cloud map generated using the original architecture.

By comparing Figure 5 and Figure 6, we can clearly observe that the improved architecture generates a larger number of point clouds. This enhancement not only excels on flat road surfaces, but also achieves a refined representation of speed bumps. This signifies that we could meet the precision requirements necessary for extracting road information. Based on this, formal experiments can be further conducted to verify the feasibility and effectiveness of this improved architecture. Figure 7 illustrates the uneven road surface within the campus used for the formal experiment, where the driver traveled in a straight line along the road at a certain speed to perform terrain construction. Figure 8 shows the point cloud map generated using the original architecture.

**Figure 9:**
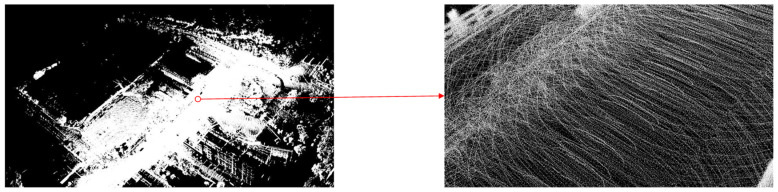
Improved architecture-generated point cloud maps.

As shown in Figure 8, the road surface information is presented at a basic level. However, the point cloud accuracy is insufficient for the extraction of road information addressed in this paper; hence, further experiments were conducted using the improved architecture. Figure 9 displays the point cloud map generated using the improved architecture.

As observed in Figure 9, the number of point clouds generated by the improved architecture was significantly greater than that of the original architecture. The point cloud count increased from 3,501,510 to 45,551,861, providing a complete depiction of the road surface details, which facilitated the extraction of road information.

## 4. Road Information Extraction Model Based on Point Cloud Library and Moving Average-like Algorithm

### 4.1. Extraction of Point Cloud Information for Front Tire Trajectories

**Figure 10:**
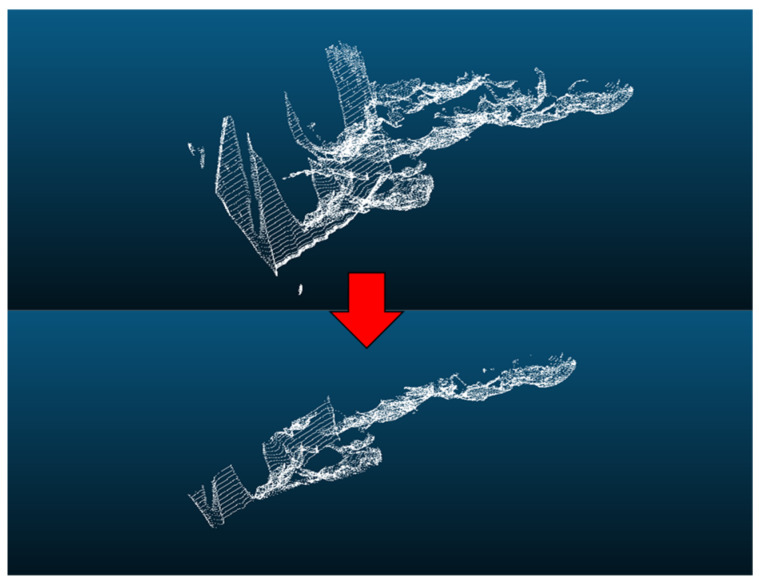
Effect of the Passthrough algorithm.

In point cloud map processing, extracting point cloud information for the front tire trajectory positions is crucial for determining the road surface elevation where the wheels are about to pass. To achieve this, this paper employed the Passthrough algorithm [32,33] from the PCL (point cloud library) to filter the point cloud information corresponding to the front tire trajectory positions. The Passthrough algorithm is a commonly used point cloud processing method that filters point cloud data based on specific conditions to extract data within a certain range. Figure 10 illustrates the effect of the Passthrough algorithm.

**Figure 11:**
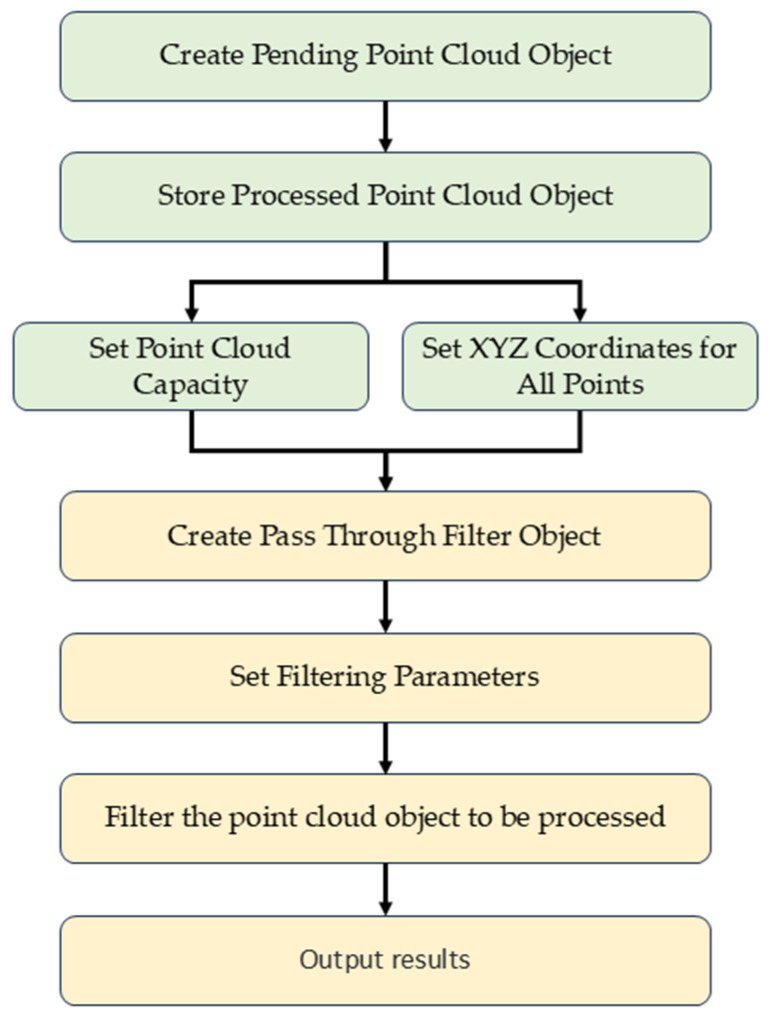
Flowchart of the Passthrough algorithm.

The process of the Passthrough algorithm is illustrated in Figure 11. The algorithm filters point cloud data by setting a range, retaining only the point cloud data that meet the specified conditions, and discarding the data that do not. Taking point cloud data in a three-dimensional space as an example, the Passthrough algorithm can impose constraints on the coordinates of points along the *x*, *y*, and *z* axes separately. By setting minimum and maximum values for each axis, a cubic or rectangular range is specified to filter the required point cloud data. This filtering method efficiently extracts the point cloud information corresponding to the front tire trajectory positions.

### 4.2. Moving Average-like Algorithm

The moving average algorithm [34] is a commonly used signal processing technique for smoothing time series data. However, the results of the moving average algorithm are influenced by historical records, whereas road information is objective and historical road data should not affect the generation of the next frame of road information. Therefore, it is necessary to eliminate the impact of historical factors in the algorithm design and develop a moving average-like algorithm suitable for road elevation information extraction. When selecting the width of the sliding window, it should be based on the tire contact area, ensuring it is neither too large nor too small, balancing both precision and efficiency. This study compared moving average-like algorithms with sliding window widths of 4 cm, 6 cm, 8 cm, and 10 cm to choose the width that provides the best results as the final algorithm.

Using a sliding window width of 4 cm as an example, the detailed algorithm design process is explained as follows:

(1) Initial grid region creation: Starting from the longitudinal distance x=0 m, first draw a grid area with a length of 0.04 m (in the x direction) and a width of 0.205 m (in the y direction), with no restriction in the z direction. In this grid, x∈(0, 0.04) m. Then, move forward by 0.01 m and draw another grid area with the same dimensions: length 0.04 m (in the x direction) and width 0.205 m (in the y direction), with no restriction in the z direction. For this grid, x ∈ (0.01, 0.05) m. Repeat this process until you reach the final grid area with a length of 0.04 m (in the x direction), and a width of 0.205 m (in the y direction), with no restriction in the z direction. For this final grid, x∈(19.96, 20) m. In the end, a total of 1997 grid areas, each 4 cm in width, will be obtained.
(2) Point assignment to grid regions: Iterate through the points and place each point into the corresponding grid areas based on its x value. A single point may fall into multiple grid areas. For example, a point with an x value of 3 cm will be included in the grid areas spanning 0~4 cm, 1~5 cm, 2~6 cm, and 3~7 cm. Therefore, this point needs to be placed into all of these overlapping grid areas simultaneously.
(3) Data storage: Create a vector container to store the x, y, and z data of the points for each grid region.
(4) Calculate averages: In a single grid, calculate the average x value and the average z value of all points within the grid. These averages represent the x and z values for that particular grid.
(5) Generate elevation information: Perform the same operation for the other grids, iterating through all grids. This will yield 1997 points with x values as the horizontal coordinates and z values as the vertical coordinates. By plotting these points and connecting them on the x–z coordinate plane, you will obtain the elevation information of the road surface.

### 4.3. Results and Discussion

Based on the terrain construction experiment, when the vehicle travels in a straight line, the trajectory equation is as follows:

$$x\in \left[0\;m,20\;m\right]$$

y∈[−0.88 m,−0.675 m]

**Figure 12:**
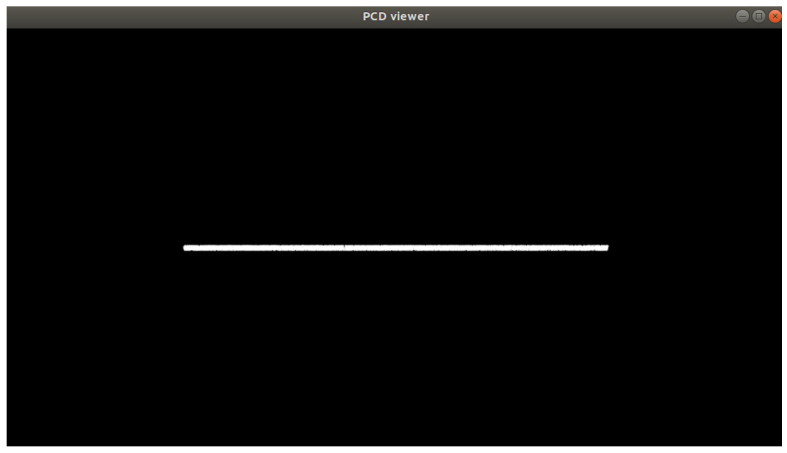
Top view of point cloud data for the front tire trajectory position.

Processing the complete point cloud map according to the preset trajectory equation, we successfully filtered out the point cloud information of the front tire trajectory position through the Passthrough algorithm. Figure 12 shows the top view of the point cloud at the front tire trajectory position after processing.

This filtered point cloud data provided essential support for the further analysis of the road surface elevation at the wheel’s path, contributing to enhancing the accuracy and reliability of road condition assessments.

Next, this paper used the moving average-like algorithm and Gaussian filter algorithm [35] to extract surface elevation information from the selected point cloud data, thereby validating the superiority of the moving average-like algorithm in extracting surface elevation information.

**Figure 13:**
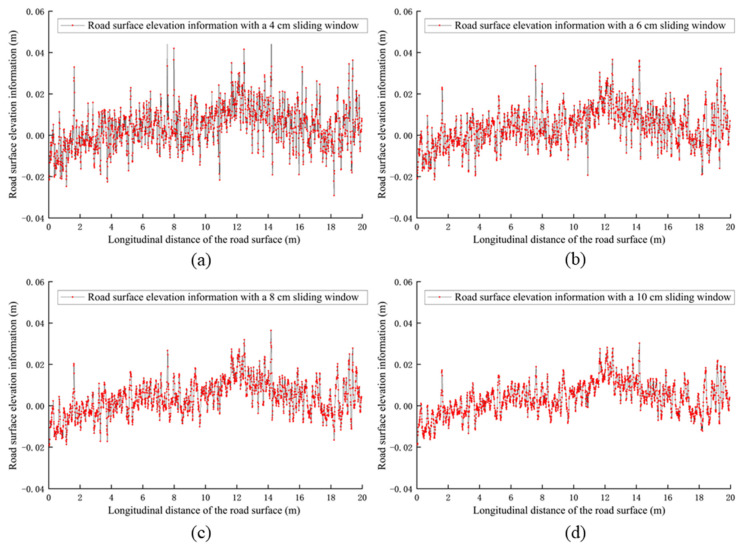
Road surface elevation information generated using moving average-like algorithm.

Figure 13 shows the road surface elevation information generated using the moving average-like algorithm with sliding window widths of 4 cm, 6 cm, 8 cm, and 10 cm. From Figure 13, it can be seen that the moving average-like algorithm preserves some high-frequency variation details, preventing the loss of important data. Additionally, as the width of the sliding window decreases, the details of the road elevation information are better presented. This algorithm optimizes the smoothing effect by using sliding windows of different widths, avoiding excessive smoothing or distortion of the data.

Generally, the larger the window width, the stronger the smoothing effect, but it also results in the loss of more local details, which reduces the consistency or accuracy of the data. A larger window averages out changes over a broader range, potentially masking smaller but important variations in the data. Additionally, for highly dynamic or rapidly changing scenarios, a larger window may lead to excessive smoothing, thus overlooking important local fluctuations or features. Therefore, reducing the sliding window width can yield more accurate road elevation information. However, a smaller window is not always better: if the window is too small, the algorithm may fail to effectively remove the noise, leading to chaotic and unreliable results. In this paper, a 4 cm sliding window was chosen, as it effectively preserved enough road detail while eliminating the interference caused by noise.

**Figure 14:**
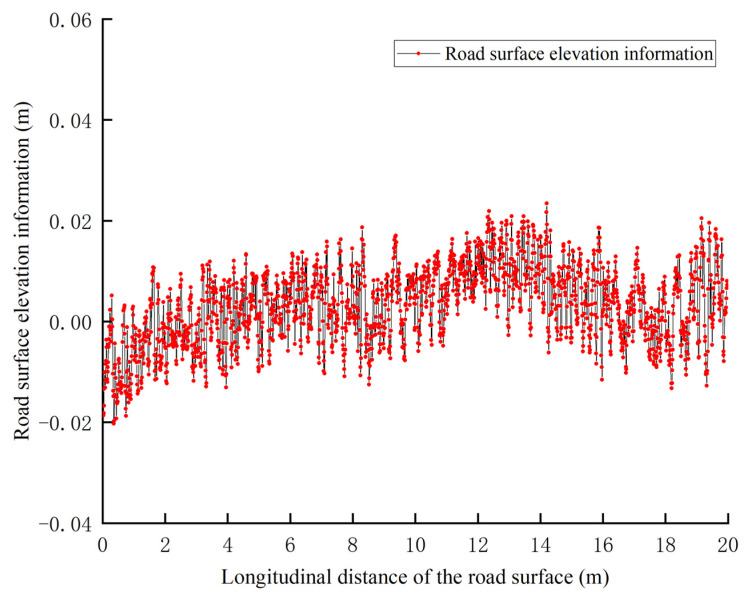
Road surface elevation information generated using Gaussian filter algorithm.

Figure 14 shows the road surface elevation information generated using the Gaussian filter algorithm. The algorithm effectively smooths the data, removes noise, and retains the overall trend of the data, making it suitable for most smoothing needs. However, by comparing it with the ground truth road elevation data, it can be observed that the algorithm may cause excessive smoothing at the edges and in detailed areas, leading to information loss, especially in the case of high-frequency information, which makes the extracted road elevation data less accurate.

After summarizing the information from Figure 13 and Figure 14 into Table 1, it can be observed that the road surface elevation data generated using the moving average algorithm with a sliding window width of 4 cm was closest to the actual road surface, achieving a congruence of 97.6%. Therefore, the road surface elevation information with the sliding window width of 4 cm, which showed the best results, was selected for the final analysis.

## 5. Conclusions

In response to challenges related to the difficulty of obtaining elevation information for the road ahead of a vehicle, this paper proposes a digital terrain construction scheme based on the fusion perception of IMU and LiDAR. This scheme establishes a terrain construction system using the fusion of IMU and LiDAR, and introduces improvements to the original framework to extract more detailed road surface point cloud information. Based on the output information from the terrain construction system, the moving average-like algorithm is designed to process the point cloud data and extract the road surface elevation information at the vehicle’s front tire trajectory. By comparing different sliding window width algorithm schemes, the optimal width was selected to ensure that the extracted road surface elevation information is more accurate and effective.

## Figures and Tables

**Table 1:** Algorithm scheme comparison.

Algorithmic Approach	Congruence with the Actual Road Surface
Sliding window width of 4 cm	97.6%
Sliding window width of 6 cm	90.3%
Sliding window width of 8 cm	82.8%
Sliding window width of 10 cm	74.5%
Gaussian filter	71.4%

## Data Availability

All data are contained within the article.
